# Crystal structure of erioflorin isolated from *Podanthus mitiqui* (L.)

**DOI:** 10.1107/S2056989017001700

**Published:** 2017-02-07

**Authors:** Cristian Paz, Leandro Ortiz, Uwe Schilde

**Affiliations:** a Universidad de La Frontera, Departamento de Ciencias Quimicas y Recursos Naturales, Avenida Francisco Salazar 01145, 4811230 Temuco, Chile; b Universidad Austral de Chile, Instituto de Ciencias Quimica, Facultad de Ciencias, Casilla 567, 5090000 Valdivia, Chile; c Universität Potsdam, Institut für Chemie, Anorganische Chemie, Karl-Liebknecht-Strasse 24-25, D-14476 Potsdam, Germany

**Keywords:** crystal structure, germacrane sesquiterpene lactone, *Podanthus mitiqui*

## Abstract

The title compound, erioflorin, C_19_H_24_O_6_ [systematic name: (1a*R*,3*S*,4*Z*,5a*R*,8a*R*,9*R*,10a*R*)-1a,2,3,5a,7,8,8a,9,10,10a-deca­hydro-3-hy­droxy-4,10a-dimethyl-8-methyl­idene-7-oxooxireno[5,6]cyclo­deca­[1,2-*b*]furan-9-yl methacrylate], is a tricyclic germacrane sesquiterpene lactone, which was isolated from *Podanthus mitiqui* (L.). The compound crystallizes in the space group *P*2_1_2_1_2_1_, and its mol­ecular structure consists of a methacrylic ester of a ten-membered ring sesquiterpenoid annelated with an epoxide and a butyrolactone. The structure is stabilized by one intramolecular C—H⋯O hydrogen bond. An O—H⋯O hydrogen bond and further C—H⋯O interactions can be observed in the packing.

## Abstract 2

The structure of erioflorin, a germacrane sesquiterpene lactone isolated from *Podanthus mitiqui*, is reported and the stereochemical features established.

## Chemical context   

*Podanthus mitiqui* (Lindl) [Asteraceae, Compositae] is an endemic plant of the Central Zone of Chile. It is an evergreen shrub that can reach up to two meters in height; its flowers are yellow or orange–yellow globose inflorescences. Previous chemical investigations of extracts isolated from the stems and leaves of *Podanthus mitiqui* revealed the presence of sesquiterpene lactones with a germacrane framework such as ovatifolin, de­acetyl­ovatifolin and arturin (Hoeneisen *et al.*, 1980 ▸) as well as erioflorin methacrylate and heliangine methacrylate (Hoeneisen *et al.*, 1981 ▸). Sesquiterpene lactones show significant anti-inflammatory, cytotoxic (Ghantous *et al.*, 2010 ▸) and anti­protozoal activities (Kaur *et al.*, 2009 ▸; Cea *et al.*, 1990 ▸; Bautista *et al.*, 2012 ▸) that make them inter­esting as attractive skeletons for drug design (for their toxic activities, see Schmidt, 1999 ▸). The natural compound erioflorin has previously been isolated from *Eriophyllum confertiflorum* (Torrance *et al.*, 1969 ▸), *Podanthus ovatifolius* (Gnecco *et al.*, 1973 ▸), *Helianthus tuberosus* (Morimoto & Oshio, 1981 ▸), *Viguiera eriophora* (Delgado *et al.*, 1982 ▸; Spring *et al.*, 2000 ▸) and *Eriophyllum lanatum* (Cea *et al.*, 1990 ▸). Now we report the title compound from *P. mitiqui*. Erioflorin has strong cytotoxic activity for the stabilization of the tumor suppressor Pdcd4 by inhibiting its inter­action with the E3-ligase β-TrCP1 and inter­feres with cell cycle progression and proliferation of tumor cells (Blees *et al.*, 2012 ▸). Herein we present the crystal structure of erioflorin in order to establish unambiguously the stereochemical features of this natural compound.

## Structural commentary   

**Figure 1:**
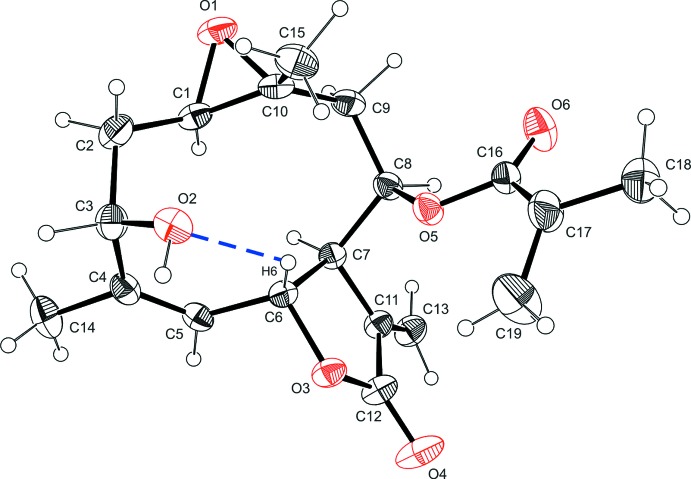
The mol­ecular structure of erioflorin with the atom labelling. Displacement ellipsoids are drawn at the 30% probability level. H atoms are shown as small spheres of arbitrary radius and hydrogen bonds as blue dashed lines.

The mol­ecule is built up from a 1,10-epoxidized ten-membered ring with hydroxyl, methyl­acryl and two methyl substituents (Fig. 1 ▸). This ring is 5,6-fused with a five-membered lactone ring with a vinyl group as substituent. The dihedral angles between the mean planes of the ten-membered ring and the lactone and the epoxide rings are 45.2 (1) and 45.7 (2)°, respectively.
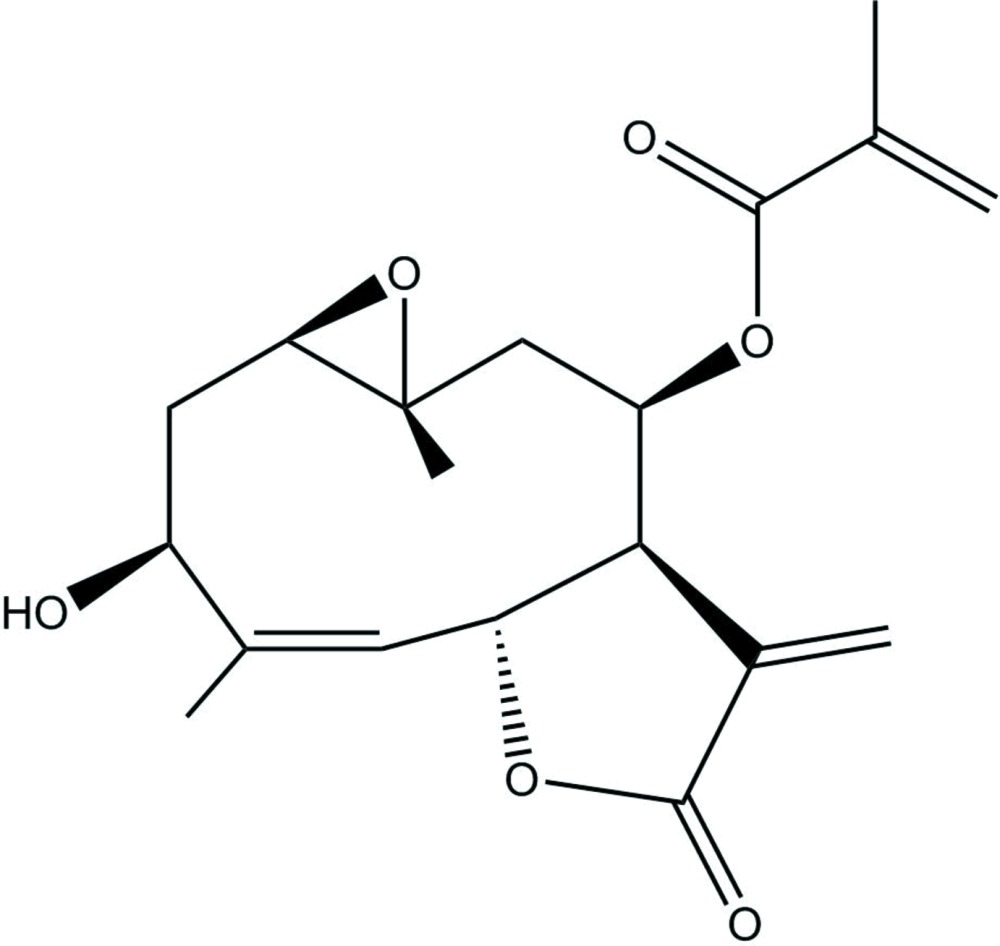


The ten-membered ring adopts an extended crown conformation with puckering amplitudes from 0.257 (3) to 0.805 (3) Å, yielding a total puckering amplitude *q* = 1.161 (3) Å and smallest displacement parameters *φ* of 23.2 (8), 252.2 (3) and 346.1 (2)°. The maximum deviation from the mean plane is 0.589 (3) Å (C3). The C—C bond lengths range from 1.474 (5) to 1.557 (4) Å. The *Z*-configured double bond is located between C4 and C5 with a bond length of 1.326 (4) Å. Some bond angles differ notably from ideal values due to the ring strain, such as C3—C4—C5 and C4—C5—C6 [125.7 (3) and 127.5 (3)°, respectively]. The bond angles within the ten-membered ring including C*sp* ^3^ atoms range from 112.0 (3)° to 125.7 (3)°. The ten-membered and the five-membered rings are *trans*-fused. The lactone ring shows a closed puckering on C6-C7 (twisted). The puckering amplitude and the smallest displacement parameter of the five-membered ring are *q* = 0.192 (3) Å and *φ* = 58.7 (9)°. With respect to the lactone ring, H6 and H7 are equatorially oriented, whereas the C6—C5 and the C7—C8 bonds are axial. The maximum deviations of the substituents from the best plane are 0.065 (6) Å (O4) and −0.323 (6) Å (C13). The 1,10-ep­oxy ring is *trans*-fused. The C8 side chain is *β* oriented as well as the C10 methyl group, whereas the C4 methyl group is *α*. The methacrylate substituent deviates from the planarity by twisting about C16—C17 [torsion angle O5—C16—C17—C19 = 28.4 (5)°]. The structure is closely related to that of di­hydro­heliangine mono­chlorido acetate (Nishikawa *et al.*, 1966 ▸). Heliangine contains dimethacrylate instead of methacrylate. Further similar compounds are eriophyllin (5-position: –AcO instead of OH; 6-position: –CH_2_OH instead of –CH_3_), eriophyllin-B (6-position: CH_2_OH; 8-position: unsubstituted) and eriophyllin-C (6-position: –CHO; 8-position: unsubstituted), which were also isolated from *Eriophyllum confertiflorum* (Torrance *et al.*, 1969 ▸). Their crystal structures are hitherto unknown. The X-ray analysis provides the relative configuration. The correct absolute configuration of the molecule was assigned to agree with the known chirality of erioflorin and is particularly based on the positions of the C6 and C7 protons as β and α, respectively, and of the methacrylate substituent as β (Torrance *et al.*, 1969 ▸; Gnecco *et al.*, 1973 ▸).

## Supra­molecular features   

**Figure 2:**
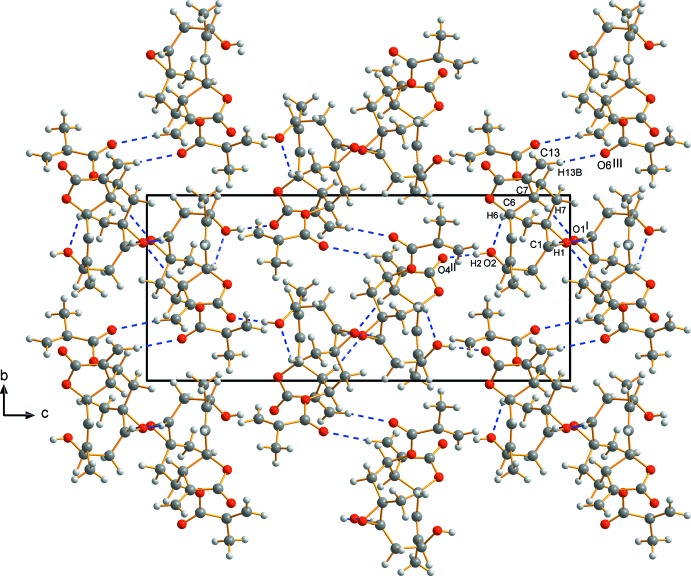
Part of the crystal structure of erioflorin, with hydrogen bonds shown as blue dashed lines. The view is along the *a* axis.

The crystal structure features infinite chains connected by hydrogen bonds. A strong O—H⋯O hydrogen bond, namely O2—H2⋯O4^ii^, running along the *c*-axis direction is formed *via* the hydroxyl group and the lactone oxo group (Fig. 2 ▸, Table 1 ▸). Furthermore, three weak C—H⋯O hydrogen bonds occur between hydrogen atoms bonded to carbon ring atoms and the oxygen atom of the same epoxide ring, running along the *a*-axis direction (C1—H1⋯O1^i^), approximately between the *a* and *b* axes (C7—H7⋯O1^i^) and along *b* (C13—H13*B*⋯O6^iii^). Non-hydrogen inter­molecular contacts are found between O2 and O4^iv^ [2.750 (4) Å; symmetry code: (iv) 1 − *x*, 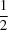 + *y*, 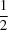 − *z*]. The unit cell contains no residual solvent-accessible voids.

## Database survey   

For structures containing the deca­hydro­oxireno[6,7]cyclo­dec-4-ene[1,2-b]furan unit, see Hull & Kennard (1978 ▸) and Bautista *et al.* (2012 ▸). For the structures of Argophyllin A, see Watson & Zabel (1982 ▸) and of Argophyllone B, see Stipanovic *et al.* (1985 ▸).

## Extraction and crystallization   

Erioflorin was isolated from *Podanthus mitiqui* collected in Concepcion, VIII Region of Chile, in February 2015 (S 36° 50′ 06.02′′ W 73° 01′ 49.36′′). Aerial parts (9.6 kg) were powdered and extracted by maceration with ethyl acetate for 3 d. The organic layer was evaporated *in vacuo* giving a crude product (250 g) which was further purified by column chromatography, giving a primary fractioning of 11 fractions (F1–F11) by using increasing polarity from hexane to ethyl acetate. F-8 (6 g) was further purified by column chromatography (silica gel 60/70–210 mesh, hexa­ne/EtOAc 1:3 *v*/*v*) giving a white solid, which was recrystallized from EtOc, affording colourless crystals suitable for X-ray diffraction analysis. M.p. (from methanol): 499–500 K. For further physical data [m.p.(methanol, ethyl acetate), α_D_, IR, ^1^H NMR] for erioflorin, see Torrance *et al.* (1969 ▸), Morimoto & Oshio (1981 ▸) and Blees *et al.* (2012 ▸).

## Refinement   

Crystal data, data collection and structure refinement details are summarized in Table 2 ▸. Hydrogen atoms were located from a difference Fourier map, but were positioned with idealized geometry and refined isotropically using a riding model with C—H = 0.97 Å (–CH_3_, allowing for rotation), C—H = 0.98 Å (–CH_2_), C—H = 0.99 Å, (–CH), C—H = 0.94 Å (=CH_2_), and *U*_iso_(H) = 1.5*U*_eq_(CH_3_) and *U*_iso_(H) = 1.2*U*_iso_(CH,CH_2_), with the exception of the O—H hydrogen atom, which was refined freely, but with *U*_iso_(H) = 1.5*U*_iso_(O).

## Supplementary Material

Crystal structure: contains datablock(s) I, global. DOI: 10.1107/S2056989017001700/zl2693sup1.cif

Structure factors: contains datablock(s) I. DOI: 10.1107/S2056989017001700/zl2693Isup2.hkl

Supporting information file. DOI: 10.1107/S2056989017001700/zl2693Isup3.cml

CCDC reference: 1530526

Additional supporting information:  crystallographic information; 3D view; checkCIF report

## Figures and Tables

**Table 1:** Hydrogen-bond geometry (Å, °)

*D*—H⋯*A*	*D*—H	H⋯*A*	*D*⋯*A*	*D*—H⋯*A*
C1—H1⋯O1^i^	0.99	2.47	3.456 (4)	173
C6—H6⋯O2	0.99	2.22	2.954 (4)	130
C7—H7⋯O1^i^	0.99	2.38	3.341 (4)	164
O2—H2⋯O4^ii^	0.85 (5)	1.90 (5)	2.750 (4)	176 (5)
C13—H13*B*⋯O6^iii^	0.94	2.57	3.493 (5)	167

Symmetry codes: (i) 
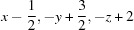
; (ii) 
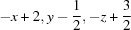
; (iii) 
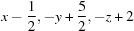
.

**Table 2:** Experimental details

Crystal data	
Chemical formula	C_19_H_24_O_6_
*M*_r_	348.38
Crystal system, space group	Orthorhombic, *P*2_1_2_1_2_1_
Temperature (K)	210
*a*, *b*, *c* (Å)	8.4709 (3), 9.8287 (3), 22.4299 (6)
*V* (Å^3^)	1867.47 (10)
*Z*	4
Radiation type	Mo *K*α
μ (mm^−1^)	0.09
Crystal size (mm)	1.02 × 0.19 × 0.06

Data collection	
Diffractometer	Stoe *IPDS* 2
Absorption correction	Integration (*X-RED*; Stoe & Cie, 2011 ▸)
*T*_min_, *T*_max_	0.585, 0.825
No. of measured, independent and observed [*I* > 2σ(*I*)] reflections	24317, 3307, 2968
*R*_int_	0.105
(sin θ/λ)_max_ (Å^−1^)	0.596

Refinement	
*R*[*F* ^2^ > 2σ(*F* ^2^)], *wR*(*F* ^2^), *S*	0.045, 0.116, 1.09
No. of reflections	3307
No. of parameters	233
H-atom treatment	H atoms treated by a mixture of independent and constrained refinement
Δρ_max_, Δρ_min_ (e Å^−3^)	0.22, −0.18

Computer programs: *X-AREA* and *X-RED* (Stoe & Cie, 2011 ▸), *SHELXS97* (Sheldrick, 2008 ▸), *ORTEP-3 for Windows* (Farrugia, 2012 ▸), *DIAMOND* (Brandenburg, 2016 ▸), *SHELXL2014* (Sheldrick, 2015 ▸) and *publCIF* (Westrip, 2010 ▸).

## supplementary crystallographic information

### Crystal data

**Table:**

C_19_H_24_O_6_	*D*_x_ = 1.239 Mg m^−^^3^
*M**_r_* = 348.38	Melting point = 498–499 K
Orthorhombic, *P*2_1_2_1_2_1_	Mo *K*α radiation, λ = 0.71073 Å
*a* = 8.4709 (3) Å	Cell parameters from 28996 reflections
*b* = 9.8287 (3) Å	θ = 1.8–25.0°
*c* = 22.4299 (6) Å	µ = 0.09 mm^−^^1^
*V* = 1867.47 (10) Å^3^	*T* = 210 K
*Z* = 4	Needle, colourless
*F*(000) = 744	1.02 × 0.19 × 0.06 mm

### Data collection

**Table:**

Stoe IPDS 2 diffractometer	3307 independent reflections
Radiation source: sealed X-ray tube	2968 reflections with *I* > 2σ(*I*)
Detector resolution: 6.67 pixels mm^-1^	*R*_int_ = 0.105
rotation method scans	θ_max_ = 25.1°, θ_min_ = 1.8°
Absorption correction: integration (X-RED; Stoe & Cie, 2011)	*h* = −10→10
*T*_min_ = 0.585, *T*_max_ = 0.825	*k* = −11→11
24317 measured reflections	*l* = −26→26

### Refinement

**Table:**

Refinement on *F*^2^	Secondary atom site location: difference Fourier map
Least-squares matrix: full	Hydrogen site location: mixed
*R*[*F*^2^ > 2σ(*F*^2^)] = 0.045	H atoms treated by a mixture of independent and constrained refinement
*wR*(*F*^2^) = 0.116	*w* = 1/[σ^2^(*F*_o_^2^) + (0.0482*P*)^2^ + 0.5108*P*] where *P* = (*F*_o_^2^ + 2*F*_c_^2^)/3
*S* = 1.09	(Δ/σ)_max_ < 0.001
3307 reflections	Δρ_max_ = 0.22 e Å^−^^3^
233 parameters	Δρ_min_ = −0.18 e Å^−^^3^
0 restraints	Extinction correction: SHELXL2014 (Sheldrick, 2015), Fc^*^=kFc[1+0.001xFc^2^λ^3^/sin(2θ)]^-1/4^
Primary atom site location: structure-invariant direct methods	Extinction coefficient: 0.019 (3)

### Special details

**Table:**

Geometry. All esds (except the esd in the dihedral angle between two l.s. planes) are estimated using the full covariance matrix. The cell esds are taken into account individually in the estimation of esds in distances, angles and torsion angles; correlations between esds in cell parameters are only used when they are defined by crystal symmetry. An approximate (isotropic) treatment of cell esds is used for estimating esds involving l.s. planes.

### Fractional atomic coordinates and isotropic or equivalent isotropic displacement parameters (Å^2^)

**Table:**

	*x*	*y*	*z*	*U*_iso_*/*U*_eq_	
C1	1.2718 (4)	0.7223 (3)	0.94856 (14)	0.0455 (7)	
H1	1.1651	0.7406	0.9646	0.055*	
C2	1.2836 (5)	0.5963 (4)	0.91111 (17)	0.0560 (9)	
H2A	1.2413	0.5195	0.9340	0.067*	
H2B	1.3954	0.5777	0.9034	0.067*	
C3	1.1965 (4)	0.6038 (3)	0.85131 (15)	0.0486 (8)	
H3	1.1997	0.5119	0.8334	0.058*	
C4	1.0244 (4)	0.6422 (3)	0.85906 (14)	0.0443 (7)	
C5	0.9680 (3)	0.7678 (3)	0.86197 (14)	0.0417 (7)	
H5	0.8582	0.7760	0.8670	0.050*	
C6	1.0579 (3)	0.8971 (3)	0.85813 (13)	0.0383 (6)	
H6	1.1702	0.8782	0.8491	0.046*	
C7	1.0446 (3)	0.9878 (3)	0.91481 (13)	0.0366 (6)	
H7	1.0075	0.9325	0.9489	0.044*	
C8	1.1992 (4)	1.0598 (3)	0.93117 (13)	0.0416 (7)	
H8	1.1719	1.1426	0.9541	0.050*	
C9	1.3134 (4)	0.9765 (4)	0.96848 (14)	0.0480 (8)	
H9A	1.4052	1.0339	0.9771	0.058*	
H9B	1.2620	0.9568	1.0066	0.058*	
C10	1.3734 (4)	0.8436 (4)	0.94334 (15)	0.0460 (8)	
C11	0.9193 (4)	1.0888 (3)	0.89732 (14)	0.0421 (7)	
C12	0.9029 (4)	1.0827 (4)	0.83196 (15)	0.0509 (8)	
C13	0.8339 (4)	1.1686 (4)	0.93126 (18)	0.0557 (9)	
H13A	0.7575	1.2258	0.9141	0.067*	
H13B	0.8491	1.1686	0.9728	0.067*	
C14	0.9170 (5)	0.5216 (4)	0.86532 (19)	0.0637 (10)	
H14A	0.9229	0.4664	0.8295	0.095*	
H14B	0.8094	0.5527	0.8710	0.095*	
H14C	0.9494	0.4678	0.8994	0.095*	
C15	1.5071 (4)	0.8528 (5)	0.89945 (19)	0.0625 (10)	
H15A	1.5415	0.7620	0.8887	0.094*	
H15B	1.5943	0.9021	0.9173	0.094*	
H15C	1.4717	0.9005	0.8640	0.094*	
C16	1.3628 (4)	1.2116 (3)	0.87355 (16)	0.0461 (7)	
C17	1.4187 (5)	1.2424 (4)	0.81157 (19)	0.0636 (10)	
C18	1.5204 (4)	1.3623 (4)	0.8052 (2)	0.0637 (10)	
H18A	1.5912	1.3680	0.8391	0.096*	
H18B	1.4555	1.4436	0.8036	0.096*	
H18C	1.5815	1.3548	0.7688	0.096*	
C19	1.3174 (7)	1.2043 (6)	0.76391 (19)	0.0893 (16)	
H19A	1.3158	1.2558	0.7286	0.107*	
H19B	1.2519	1.1275	0.7677	0.107*	
O1	1.3989 (3)	0.7432 (3)	0.99078 (11)	0.0602 (7)	
O2	1.2835 (3)	0.6918 (3)	0.81346 (11)	0.0502 (6)	
H2	1.245 (5)	0.681 (5)	0.779 (2)	0.075*	
O3	0.9867 (3)	0.9803 (2)	0.80995 (9)	0.0466 (6)	
O4	0.8276 (4)	1.1601 (3)	0.80088 (13)	0.0800 (10)	
O5	1.2672 (3)	1.1032 (2)	0.87473 (10)	0.0472 (6)	
O6	1.4016 (4)	1.2734 (3)	0.91723 (13)	0.0680 (8)	

### Atomic displacement parameters (Å^2^)

**Table:**

	*U*^11^	*U*^22^	*U*^33^	*U*^12^	*U*^13^	*U*^23^
C1	0.0439 (16)	0.0556 (19)	0.0370 (15)	0.0101 (15)	−0.0056 (13)	0.0065 (14)
C2	0.060 (2)	0.0514 (18)	0.057 (2)	0.0157 (18)	−0.0042 (17)	0.0038 (17)
C3	0.0514 (18)	0.0456 (16)	0.0488 (19)	0.0022 (15)	0.0046 (15)	−0.0048 (15)
C4	0.0465 (16)	0.0476 (17)	0.0389 (16)	−0.0089 (14)	0.0022 (14)	−0.0047 (14)
C5	0.0334 (13)	0.0507 (17)	0.0411 (16)	−0.0036 (13)	0.0012 (12)	−0.0064 (15)
C6	0.0364 (14)	0.0408 (15)	0.0377 (15)	0.0020 (12)	−0.0030 (12)	0.0026 (13)
C7	0.0344 (14)	0.0395 (15)	0.0358 (14)	0.0005 (12)	0.0007 (11)	0.0006 (12)
C8	0.0416 (16)	0.0474 (16)	0.0358 (15)	−0.0056 (14)	−0.0033 (12)	−0.0008 (13)
C9	0.0445 (17)	0.061 (2)	0.0380 (15)	−0.0040 (16)	−0.0091 (13)	−0.0004 (15)
C10	0.0368 (16)	0.061 (2)	0.0407 (17)	0.0054 (14)	−0.0096 (13)	0.0035 (14)
C11	0.0431 (17)	0.0443 (16)	0.0389 (15)	0.0035 (14)	−0.0012 (13)	0.0028 (13)
C12	0.0521 (19)	0.059 (2)	0.0420 (17)	0.0135 (17)	−0.0024 (14)	0.0072 (16)
C13	0.056 (2)	0.057 (2)	0.055 (2)	0.0140 (17)	0.0025 (16)	0.0002 (17)
C14	0.072 (2)	0.056 (2)	0.063 (2)	−0.0208 (19)	0.0096 (19)	−0.0086 (19)
C15	0.0373 (18)	0.085 (3)	0.065 (2)	−0.0032 (18)	0.0051 (16)	−0.003 (2)
C16	0.0453 (17)	0.0383 (15)	0.0547 (19)	−0.0019 (13)	−0.0024 (14)	−0.0011 (16)
C17	0.064 (2)	0.065 (2)	0.063 (2)	−0.019 (2)	0.0036 (18)	0.013 (2)
C18	0.049 (2)	0.057 (2)	0.085 (3)	0.0022 (17)	0.014 (2)	0.000 (2)
C19	0.110 (4)	0.114 (4)	0.045 (2)	−0.046 (3)	0.005 (2)	0.005 (2)
O1	0.0578 (14)	0.0738 (16)	0.0491 (14)	0.0128 (13)	−0.0181 (11)	0.0102 (12)
O2	0.0420 (12)	0.0630 (14)	0.0458 (12)	0.0000 (11)	0.0056 (10)	−0.0082 (11)
O3	0.0506 (12)	0.0543 (13)	0.0349 (11)	0.0070 (11)	−0.0031 (9)	0.0019 (10)
O4	0.090 (2)	0.097 (2)	0.0535 (16)	0.0422 (18)	−0.0130 (15)	0.0130 (16)
O5	0.0506 (13)	0.0508 (12)	0.0402 (11)	−0.0137 (11)	−0.0016 (9)	0.0038 (10)
O6	0.0806 (18)	0.0558 (14)	0.0675 (17)	−0.0177 (14)	0.0062 (14)	−0.0152 (14)

### Geometric parameters (Å, º)

**Table:**

C1—O1	1.449 (4)	C10—O1	1.467 (4)
C1—C10	1.475 (5)	C10—C15	1.503 (5)
C1—C2	1.499 (5)	C11—C13	1.311 (5)
C1—H1	0.9900	C11—C12	1.474 (5)
C2—C3	1.532 (5)	C12—O4	1.213 (4)
C2—H2A	0.9800	C12—O3	1.327 (4)
C2—H2B	0.9800	C13—H13A	0.9400
C3—O2	1.418 (4)	C13—H13B	0.9400
C3—C4	1.516 (5)	C14—H14A	0.9700
C3—H3	0.9900	C14—H14B	0.9700
C4—C5	1.326 (4)	C14—H14C	0.9700
C4—C14	1.501 (5)	C15—H15A	0.9700
C5—C6	1.484 (4)	C15—H15B	0.9700
C5—H5	0.9400	C15—H15C	0.9700
C6—O3	1.483 (3)	C16—O6	1.199 (4)
C6—C7	1.557 (4)	C16—O5	1.338 (4)
C6—H6	0.9900	C16—C17	1.499 (5)
C7—C11	1.505 (4)	C17—C19	1.421 (6)
C7—C8	1.533 (4)	C17—C18	1.467 (5)
C7—H7	0.9900	C18—H18A	0.9700
C8—O5	1.455 (4)	C18—H18B	0.9700
C8—C9	1.519 (4)	C18—H18C	0.9700
C8—H8	0.9900	C19—H19A	0.9400
C9—C10	1.511 (5)	C19—H19B	0.9400
C9—H9A	0.9800	O2—H2	0.85 (5)
C9—H9B	0.9800		
			
O1—C1—C10	60.2 (2)	H9A—C9—H9B	107.1
O1—C1—C2	115.7 (3)	O1—C10—C1	59.0 (2)
C10—C1—C2	125.7 (3)	O1—C10—C15	113.9 (3)
O1—C1—H1	114.5	C1—C10—C15	122.7 (3)
C10—C1—H1	114.5	O1—C10—C9	111.1 (3)
C2—C1—H1	114.5	C1—C10—C9	118.2 (3)
C1—C2—C3	114.7 (3)	C15—C10—C9	116.4 (3)
C1—C2—H2A	108.6	C13—C11—C12	123.3 (3)
C3—C2—H2A	108.6	C13—C11—C7	129.2 (3)
C1—C2—H2B	108.6	C12—C11—C7	107.4 (3)
C3—C2—H2B	108.6	O4—C12—O3	122.9 (3)
H2A—C2—H2B	107.6	O4—C12—C11	126.6 (3)
O2—C3—C4	114.6 (3)	O3—C12—C11	110.5 (3)
O2—C3—C2	107.6 (3)	C11—C13—H13A	120.0
C4—C3—C2	112.0 (3)	C11—C13—H13B	120.0
O2—C3—H3	107.4	H13A—C13—H13B	120.0
C4—C3—H3	107.4	C4—C14—H14A	109.5
C2—C3—H3	107.4	C4—C14—H14B	109.5
C5—C4—C14	120.8 (3)	H14A—C14—H14B	109.5
C5—C4—C3	125.7 (3)	C4—C14—H14C	109.5
C14—C4—C3	113.4 (3)	H14A—C14—H14C	109.5
C4—C5—C6	127.5 (3)	H14B—C14—H14C	109.5
C4—C5—H5	116.2	C10—C15—H15A	109.5
C6—C5—H5	116.2	C10—C15—H15B	109.5
O3—C6—C5	107.8 (2)	H15A—C15—H15B	109.5
O3—C6—C7	104.5 (2)	C10—C15—H15C	109.5
C5—C6—C7	113.9 (2)	H15A—C15—H15C	109.5
O3—C6—H6	110.2	H15B—C15—H15C	109.5
C5—C6—H6	110.2	O6—C16—O5	123.6 (3)
C7—C6—H6	110.2	O6—C16—C17	124.7 (3)
C11—C7—C8	111.1 (2)	O5—C16—C17	111.7 (3)
C11—C7—C6	102.5 (2)	C19—C17—C18	119.6 (4)
C8—C7—C6	113.4 (2)	C19—C17—C16	117.0 (3)
C11—C7—H7	109.9	C18—C17—C16	115.9 (4)
C8—C7—H7	109.9	C17—C18—H18A	109.5
C6—C7—H7	109.9	C17—C18—H18B	109.5
O5—C8—C9	112.6 (3)	H18A—C18—H18B	109.5
O5—C8—C7	105.4 (2)	C17—C18—H18C	109.5
C9—C8—C7	115.3 (3)	H18A—C18—H18C	109.5
O5—C8—H8	107.7	H18B—C18—H18C	109.5
C9—C8—H8	107.7	C17—C19—H19A	120.0
C7—C8—H8	107.7	C17—C19—H19B	120.0
C10—C9—C8	118.3 (3)	H19A—C19—H19B	120.0
C10—C9—H9A	107.7	C1—O1—C10	60.8 (2)
C8—C9—H9A	107.7	C3—O2—H2	106 (3)
C10—C9—H9B	107.7	C12—O3—C6	111.4 (2)
C8—C9—H9B	107.7	C16—O5—C8	119.4 (2)
			
O1—C1—C2—C3	−155.0 (3)	C8—C9—C10—O1	−146.5 (3)
C10—C1—C2—C3	−84.4 (4)	C8—C9—C10—C1	−81.3 (4)
C1—C2—C3—O2	72.5 (4)	C8—C9—C10—C15	80.9 (4)
C1—C2—C3—C4	−54.4 (4)	C8—C7—C11—C13	−76.3 (4)
O2—C3—C4—C5	−36.3 (5)	C6—C7—C11—C13	162.3 (4)
C2—C3—C4—C5	86.7 (4)	C8—C7—C11—C12	104.9 (3)
O2—C3—C4—C14	145.4 (3)	C6—C7—C11—C12	−16.5 (3)
C2—C3—C4—C14	−91.6 (4)	C13—C11—C12—O4	10.6 (7)
C14—C4—C5—C6	178.4 (3)	C7—C11—C12—O4	−170.5 (4)
C3—C4—C5—C6	0.3 (6)	C13—C11—C12—O3	−170.9 (3)
C4—C5—C6—O3	125.5 (3)	C7—C11—C12—O3	7.9 (4)
C4—C5—C6—C7	−119.1 (4)	O6—C16—C17—C19	−153.5 (5)
O3—C6—C7—C11	19.0 (3)	O5—C16—C17—C19	28.4 (5)
C5—C6—C7—C11	−98.4 (3)	O6—C16—C17—C18	−3.8 (6)
O3—C6—C7—C8	−100.9 (3)	O5—C16—C17—C18	178.1 (3)
C5—C6—C7—C8	141.8 (3)	C2—C1—O1—C10	118.1 (3)
C11—C7—C8—O5	−74.8 (3)	C15—C10—O1—C1	−115.1 (3)
C6—C7—C8—O5	40.0 (3)	C9—C10—O1—C1	111.1 (3)
C11—C7—C8—C9	160.4 (3)	O4—C12—O3—C6	−176.3 (4)
C6—C7—C8—C9	−84.8 (3)	C11—C12—O3—C6	5.2 (4)
O5—C8—C9—C10	−61.5 (4)	C5—C6—O3—C12	105.8 (3)
C7—C8—C9—C10	59.5 (4)	C7—C6—O3—C12	−15.7 (3)
C2—C1—C10—O1	−101.8 (3)	O6—C16—O5—C8	3.0 (5)
O1—C1—C10—C15	100.1 (3)	C17—C16—O5—C8	−178.9 (3)
C2—C1—C10—C15	−1.7 (5)	C9—C8—O5—C16	−80.4 (3)
O1—C1—C10—C9	−98.9 (3)	C7—C8—O5—C16	153.1 (3)
C2—C1—C10—C9	159.3 (3)		

### Hydrogen-bond geometry (Å, º)

**Table:**

*D*—H···*A*	*D*—H	H···*A*	*D*···*A*	*D*—H···*A*
C1—H1···O1^i^	0.99	2.47	3.456 (4)	173
C6—H6···O2	0.99	2.22	2.954 (4)	130
C7—H7···O1^i^	0.99	2.38	3.341 (4)	164
O2—H2···O4^ii^	0.85 (5)	1.90 (5)	2.750 (4)	176 (5)
C13—H13*B*···O6^iii^	0.94	2.57	3.493 (5)	167

Symmetry codes: (i) *x*−1/2, −*y*+3/2, −*z*+2; (ii) −*x*+2, *y*−1/2, −*z*+3/2; (iii) *x*−1/2, −*y*+5/2, −*z*+2.
